# Randomised Controlled Trial of Joint Crisis Plans to Reduce Compulsory Treatment for People with Psychosis: Economic Outcomes

**DOI:** 10.1371/journal.pone.0074210

**Published:** 2013-11-25

**Authors:** Barbara Barrett, Waquas Waheed, Simone Farrelly, Max Birchwood, Graham Dunn, Clare Flach, Claire Henderson, Morven Leese, Helen Lester, Max Marshall, Diana Rose, Kim Sutherby, George Szmukler, Graham Thornicroft, Sarah Byford

**Affiliations:** 1 Health Service and Population Research Department, Institute of Psychiatry, King's College London, London, United Kingdom; 2 Division of Psychiatry, School of Medicine, University of Manchester, Manchester, United Kingdom; 3 Department of Psychology, University of Birmingham, Birmingham, United Kingdom; 4 Institute of Population Health, University of Manchester, Manchester, United Kingdom; 5 School of Health and Population Sciences, University of Birmingham, Birmingham, United Kingdom; Federal University of Rio de Janeiro, Brazil

## Abstract

**Background:**

Compulsory admission to psychiatric hospitals may be distressing, disruptive to patients and families, and associated with considerable cost to the health service. Improved patient experience and cost reductions could be realised by providing cost-effective crisis planning services.

**Methods:**

Economic evaluation within a multi-centre randomised controlled trial comparing Joint Crisis Plans (JCP) plus treatment as usual (TAU) to TAU alone for patients aged over 16, with at least one psychiatric hospital admission in the previous two years and on the Enhanced Care Programme Approach register. JCPs, containing the patient's treatment preferences for any future psychiatric emergency, are a form of crisis intervention that aim to mitigate the negative consequences of relapse, including hospital admission and use of coercion. Data were collected at baseline and 18-months after randomisation. The primary outcome was admission to hospital under the Mental Health Act. The economic evaluation took a service perspective (health, social care and criminal justice services) and a societal perspective (additionally including criminal activity and productivity losses).

**Findings:**

The addition of JCPs to TAU had no significant effect on compulsory admissions or total societal cost per participant over 18-months follow-up. From the service cost perspective, however, evidence suggests a higher probability (80%) of JCPs being the more cost-effective option. Exploration by ethnic group highlights distinct patterns of costs and effects. Whilst the evidence does not support the cost-effectiveness of JCPs for White or Asian ethnic groups, there is at least a 90% probability of the JCP intervention being the more cost-effective option in the Black ethnic group.

**Interpretation:**

The results by ethnic group are sufficiently striking to warrant further investigation into the potential for patient gain from JCPs among black patient groups.

**Trial Registration:**

Current Controlled Trials ISRCTN11501328

## Introduction

The number of patients admitted on a compulsory basis to psychiatric hospitals in England and Wales increased by over 50% in the decade to 1995 and by 13% during the decade up to 2010/11 [1]. As well as being distressing and disruptive to patients and their families, admission to psychiatric hospitals is associated with considerable costs to the health service [2]. A recent King's Fund report suggests that savings, mainly in the form of reduced inpatient costs, could be realised by expanding the use of crisis intervention and early intervention services [3].

As described in our companion paper [4], a Joint Crisis Plan (JCP) aims to empower mental health patients whilst facilitating early detection and treatment of relapse [5]–[7]. Formulated by the patient in collaboration with staff, a JCP contains the patient's treatment preferences for any future psychiatric emergency, when he or she may be too unwell to express clear views. A JCP is a form of crisis intervention that aims to mitigate some of the negative consequences of relapse, including admission to hospital, use of coercion in the form of the Mental Health Act (MHA), and associated costs [8].

An exploratory randomised trial of JCPs found that for the JCP group compared to the control group, use of the MHA over the 15-month follow-up was significantly lower (13% versus 27%; p = 0·028), mean number of days compulsorily detained was significantly lower (14 versus 31 bed-days; p = 0·04) and overall bed-days were lower, although not significantly so (32 versus 36 bed-days; p = 0·15) [8], [9]. An economic evaluation found that the JCP intervention had a 78% probability of being more cost-effective than the control condition at reducing the proportion of patients admitted to hospital [9].

In this paper we report the results of an economic evaluation carried out alongside a multi-centre randomised controlled trial of JCPs plus treatment as usual (TAU) compared to TAU alone [4]. The primary clinical analysis, reported in our companion paper, explored whether JCPs significantly reduced the proportion of patients detained or treated under a section of the MHA over an 18-month follow-up period, and found no significant difference compared to the TAU group [4]. In such situations, traditional reliance on arbitrary decision rules regarding statistical significance would, more likely than not, result in the rejection of JCPs and support the continuation of usual care. However, such traditions are being increasingly criticised as less relevant in a decision-making context [10], [11]. Instead, it is argued that the decision to adopt one intervention over another, or to add an intervention to existing services, should be based on the expected cost-effectiveness of the intervention, or the probability of making the correct decision given the data available, which is the approach taken here.

The study was also designed and powered to analyse the effectiveness of the JCP in reducing the use of the Mental Health Act for Black (Black Caribbean and Black African) service users (12), and therefore we also report here the cost-effectiveness of JCPs by ethnic group.

## Methods

The protocol for this trial and supporting CONSORT checklist are available as supporting information; see Protocol S1 and Checklist S1.

### Ethics statement

The trial received ethical approval from King's College Hospital Research Ethics Committee and is registered with Current Controlled Trials ISRCTN11501328.

### Aims

The aim of the study was to explore the relative costs and cost-effectiveness of JCPs plus TAU (JCP group) compared to TAU alone (control group) over an 18-month follow-up period for patients with a history of relapsing psychotic illness.

### Study design and participants

Full details of the trial are reported in our protocol [12] and clinical results paper [4]. In brief, the study design was an individual level, single-blind, randomised controlled trial. Participants were recruited between August 2008 and March 2010, and followed up 18-months after randomisation. Patients were eligible if they had a history of relapsing psychotic illness, were over 16, had at least one admission to a psychiatric hospital in the previous two years, and were registered on the Enhanced Care Programme Approach (i.e., had complex needs) [13]. Patients were excluded if they were subject to a section of the MHA, to reduce the likelihood of perceived pressure to participate. Patients giving written consent for participation were recruited from generic and specialist community mental health teams within three geographical areas in four English mental health trusts: Birmingham and Solihull Mental Health Foundation Trust; Lancashire Care NHS Foundation Trust; Manchester Mental Health and Social Care Trust; and South London and Maudsley NHS Foundation Trust. Recruitment was facilitated by the provision of culturally adapted study information, consent forms and assessments. Capacity was informally assessed by trained research assistants and confirmed where necessary with the care coordinator or responsible clinician. Due to the nature of the intervention that required their active participation, if there were concerns that the participant to did not have capacity to consent, they were not recruited to the trial.

### Sample Size

A sample of 270 in each arm was chosen so that, after allowing for 15% loss to follow up and using a significance level of 0·05, the following could be detected: a reduction by half in the proportion overall admitted under the MHA with 90% power (from 30% to 15%); a proportionate reduction for a pre-specified subgroup of interest, Black patients, with 80% power (from 40% to 20%), assuming that the overall sample would yield a subsample of 91 Black patients per arm.

### Randomisation and masking

After baseline assessment, participants were stratified by site (Birmingham, London, and Lancashire/Manchester) and randomly allocated to JCP or control group using permuted blocks of randomly varying block size, with equal allocation to the two arms. Investigators and research assessors were masked to allocation.

### Interventions

The JCP intervention, described in detail elsewhere [5], [6], [8], was delivered by senior mental health nurses and involved participants in the JCP group being invited to attend two meetings, organised by a JCP facilitator, in order to generate a JCP. At the first meeting, the facilitator introduced the participant and their care coordinator to the principles of joint crisis planning and described the JCP menu – a list of suggestions that participants might want to include in their JCP. The second meeting took place at least one week later and was attended by the participant, their psychiatrist, the facilitator and the care coordinator. Participants could also invite a friend or relative. At this meeting the JCP was finalised and subsequently disseminated to the psychiatrist, care co-ordinator, and anyone else nominated by the participant. Nine months later the facilitator re-contacted the participant to ask if they wanted to update the plan. JCP facilitators received one week of training and weekly supervision. Both groups received current standard care from local community mental health teams which, as a part of the Care Programme Approach, includes the need for patients to receive written copies of their care plan including a ‘crisis contingency plan’ [13].

### Outcome measures

Data were collected at baseline and 18-months after randomisation. The primary clinical and economic outcome was admission to hospital under the MHA, which was gathered from and validated between the following sources: case notes, the local Patient Administration System, MHA Office data, and interviews of patients and care co-ordinators.

### Economic data

The economic evaluation took both a service perspective, including all hospital, community health and social care, and criminal justice sector services, and a societal perspective, additionally including the cost of criminal activity and productivity losses (days off work due to illness).

Economic data were collected using the Adult Service Use Schedule (AD-SUS), an interview-based, guided patient self-report measure designed by the authors (SB) on the basis of data from previous studies of service use in adult mental health populations [9], [14], [15]. The AD-SUS was completed at baseline for the 3 months preceding randomisation and at 18-month follow-up for the period since randomisation. To improve accuracy, interview data were supplemented by information from computerised hospital records at each site for mental health hospital admissions and community mental health services. Data on staff input into the development of each individual JCP were collected from JCP facilitator records.

Unit costs, for the financial year 2009–10, were applied to each item of service reported. Costs and outcomes falling within the second year were not discounted because the single follow-up point at 18-months post randomisation did not allow costs that fall within the first 12 months to be separated from those that occurred subsequently. Trust-specific costs for NHS hospital contacts were sourced from NHS Reference Costs and community health and social service costs were taken from national publications [16], [17]. The cost of medications was calculated using the British National Formulary [18]. Contacts with criminal justice agencies were costed using national publications and the charges used by professionals for work completed [19]. Where necessary, unit costs were inflated to 2009–10 using the Hospital and Community Health Services inflation indices or the Retail Price Index as appropriate [17]. Productivity losses were costed using the human capital approach, which involves multiplying days off work due to illness by the individual's salary [20] and explored further in sensitivity analysis.

The cost of the intervention sessions were estimated using a bottom-up, micro costing approach. First, the average salary costs were estimated for the facilitators using the mid-point from the appropriate Agenda for Change salary scale and adding on-costs of employer National Insurance and pension contributions. Next, overhead costs were added to reflect the facilitators working in a community setting. Indirect overhead costs included administrative and managerial support costs and capital overhead costs included the cost of office space. Total salary and overhead costs were then divided by the average number of working hours per year, to calculate the cost per hour. As well as time in direct contact with participants, facilitators spent time arranging the meetings and in other preparation activities such as training and administration. We therefore asked each facilitator to complete a short survey asking how long they spent on these activities in a typical week. The ratio of direct to indirect client contact that resulted from this survey was used to calculate the cost per hour of direct client contact. In addition, account was taken of the fact that the meetings were also attended by the participant's care-co-ordinator and consultant. It is likely if the JCP intervention were to be rolled out into mainstream practice the facilitators would be able to undertake the meetings more frequently, the impact of possible changes in working practices was explored in sensitivity analyses.

### Statistical Methods

All analyses were carried out in Stata version 10 on an intention to treat basis using a statistical analysis plan drawn up prior to data analysis. The primary outcome, proportion of participants admitted to hospital under a compulsory section, was compared between randomisation groups using chi-squared tests and logistic regression and adjusted for site (Birmingham, Lancashire/Manchester, and London) and the patient rated Working Alliance Inventory (WAIC) [21], which was found to be associated with having missing data [4].

All economic analyses were adjusted for site and baseline costs and complete case analysis was used. Total costs were compared using t-tests with confidence intervals for mean differences estimated using non-parametric bootstrapping and ordinary least squares regression for adjusted analysis. Although the cost data were skewed, the advantage of this approach is the ability to make inferences about the arithmetic mean [22].

Cost-effectiveness was explored through the calculation of incremental cost-effectiveness ratios (ratio of the additional costs to the additional effects of the JCP group in comparison to the control group) [23]. Non-parametric bootstrapping (repeat re-sampling) was used to generate a joint distribution of incremental mean costs and effects for the two groups. These distributions were used to calculate the probability that each treatment is the optimal choice (the more cost-effective) for a range of maximum monetary values (ceiling ratio) a decision-maker might be willing to pay for a 1% reduction in the proportion of participants admitted to hospital under a compulsory section. Cost-effectiveness acceptability curves were generated by plotting these probabilities for a range of possible values of the ceiling ratio [24]. These curves incorporate the uncertainty that exists around the estimates of mean costs and effects as a result of sampling variation and uncertainty regarding the maximum cost-effectiveness ratio that a decision-maker would consider acceptable.

Analyses were repeated for the three main ethnic groups recruited into the study: White (62%), Black/Black British (22%) and Asian/Asian British (10%). The remaining 6% of the population, classified as mixed or other, were excluded from this part of the analysis.

### Sensitivity analyses

A number of one-way sensitivity analyses of costs were carried out. Firstly, as described above, productivity losses were costed using the human capital approach, which involves multiplying days off work due to illness by the individual's salary. A number of commentators have argued that this approach is limited since it tends to overestimate productivity losses by ignoring, for example, the ability to replace workers from the pool of unemployed people or for other members of staff to provide cover for the absent member of staff [25]. Given these limitations, productivity losses were reduced to zero to explore the full range from the maximum losses (human capital approach) to a minimum of zero (where all productivity losses are adequately covered by other staff members, for example).

Secondly, we explored the possibility that if the JCP intervention were to be rolled out into mainstream practice, the facilitators would be able to undertake more meetings per week, thus reducing the cost of each hour of face-to-face contact. In the trial, facilitators attended approximately two face-to-face JCP meetings per week. This was increased to four meetings per week, with a subsequent reduction in the cost per hour of face-to-face contact from £844 to £579.

## Results

### Participants

**Figure 1:**
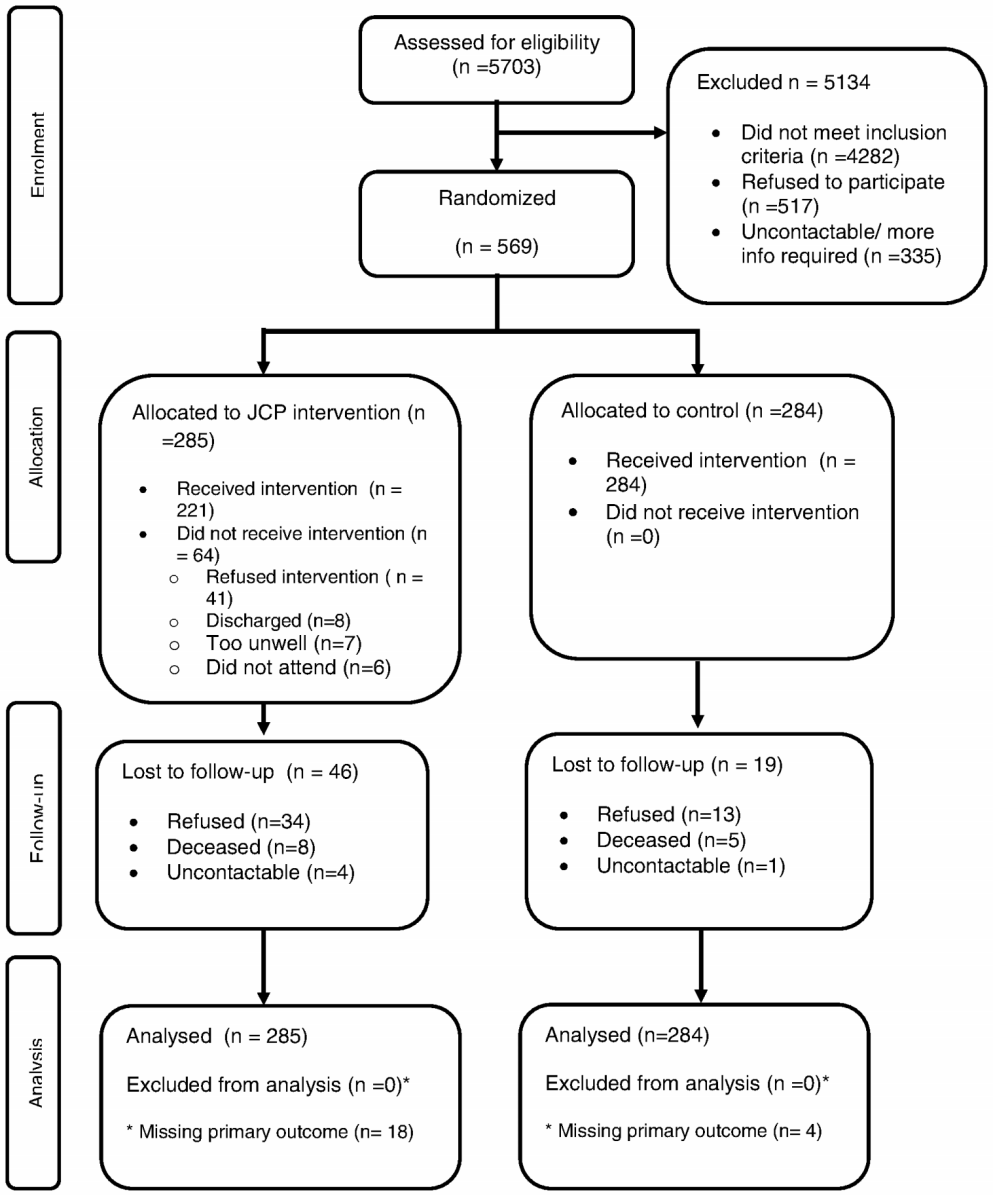
CONSORT diagram.

569 participants from 64 mental health teams were randomised to JCP (n = 285) or control group (n = 284), see CONSORT diagram in Figure 1. The sample, reported in detail in our companion paper [4], was evenly divided by gender and was aged 40 on average; 44% lived alone; 62% were White, 22% Black (African-Caribbean, Black British, Black), 10% Asian; 27% had no formal educational qualifications. Diagnoses included schizophrenia spectrum disorders (74%) and affective disorders (26%). All participants had been admitted to hospital in the previous 2 years, with a mean number of 1·5 admissions and median duration of 59 days. There were no substantial differences by randomisation arm in any of these baseline characteristics [4].

Data on the primary outcome were missing for 22 of the 569 participants (4%). A further 43 had no follow-up interview, giving 65 (11%) in total with missing data. Those with missing data were similar to those with data, except that those with missing data had significantly worse self-rated therapeutic relationship (WAIC) scores (18·6 versus 15·8; p = 0·043) and were more likely to be in the intervention arm (n = 18, 6%) versus the control arm (n = 4, 1%) [4].

### Outcomes

There was no significant treatment effect for the primary outcome with 105 compulsory admissions recorded amongst participants over the 18-month follow-up period, 49 (18%) in the JCP group and 56 (20%) in the control group (odds ratio 0·90, 95% CI 0·59 to 1·38, p = 0·63). There were no significant treatment effects for any other admissions outcomes, although there was evidence for improved therapeutic relationships in the intervention arm, described in detail in the companion paper [4].

### Costs

Service use, criminal activity and productivity losses over the 18-month follow-up period, (Table 1), were similar between the two groups and the addition of the JCP did not lead to significantly higher service costs (including JCP intervention, health and social care, accommodation and criminal justice services) or societal costs (including include all service costs plus the costs of crimes and lost employment). Total costs are summarised in Table 2. Costs were lower in the JCP group from the service perspective, but not significantly so (£17,233 per participant versus £19,217 in the control group; p = 0·414), and no different from the societal perspective (£22,501 versus £22,851; p = 0·902).

**Table 1:** Mean resource-use over the 18-month follow-up period.

	JCP group (N = 240) Mean (SD)	TAU group (N = 264) Mean (SD)
Service provided accommodation (months)	2·0 (5·4)	1·9 (5·1)
Inpatient stay - psychiatric (nights)	18·7 (53·0)	22·9 (61·8)
Inpatient stay - medical (nights)	0·9 (3·2)	0·6 (1·8)
Outpatient appointments (number)	3·0 (17·2)	2·2 (14·8)
Accident and emergency (attendances)	0·9 (3·2)	0·6 (1·8)
General practitioner (contacts)	6·6 (8·2)	7·3 (10·3)
Professions allied to medicine (contacts)	2·7 (6·6)	3·0 (17·9)
Community mental health - care team (contacts)	64·2 (60·1)	62·1 (63·1)
Community mental health - other (contacts)	2·4 (12·5)	1·4 (8·3)
Social work/housing/advice (contacts)	2·0 (10·1)	0·6 (4·7)
Home help (contacts)	7·8 (42·2)	5·5 (37·9)
Day care/drop-in (sessions)	9·1 (45·4)	14·4 (56·2)
Prison/police custody (nights/contacts)	2·1 (27·2)	2·6 (22·6)
Criminal justice professionals (contacts)	2·0 (7·2)	1·4 (5·1)
Crimes victim (number)	0·0 (0·1)	0·0 (0·0)
Crimes perpetrator (number)	22·4 (101·3)	17·7 (98·8)
Absence from work (hours)	6·0 (40·5)	9·7 (71·2)

**Table 2:** Total costs over the 18-month follow-up period; Mean, £.

	JCP group (N = 240) Mean (SD)	TAU group (N = 264) Mean (SD)	Mean difference	95% CI*	p-value*
JCP intervention	224 (367)	0 (0)			
Health and social care	13,756 (17,953)	15,744 (25,578)			
Accommodation	2,892 (9,249)	2,946 (9,006)			
Criminal justice services	351 (3,033)	527 (4,586)			
Total service costs#	17,223 (21,013)	19,217 (28,133)	−1,994	−5,733 to 2,248	0·414
Crimes	5,262 (17,220)	3,540 (13,684)			
Employment	16 (135)	94 (103)			
Total societal costs∧	22,501 (28,103)	22,851 (34,532)	−350	−4,727 to 5,404	0·902

* Adjusted for baseline costs and study site.

# Total service costs include JCP intervention, health and social care, accommodation and criminal justice services.

∧ Total societal costs include all service costs plus the costs of crimes and lost employment.

### Cost-effectiveness

**Figure 2:**
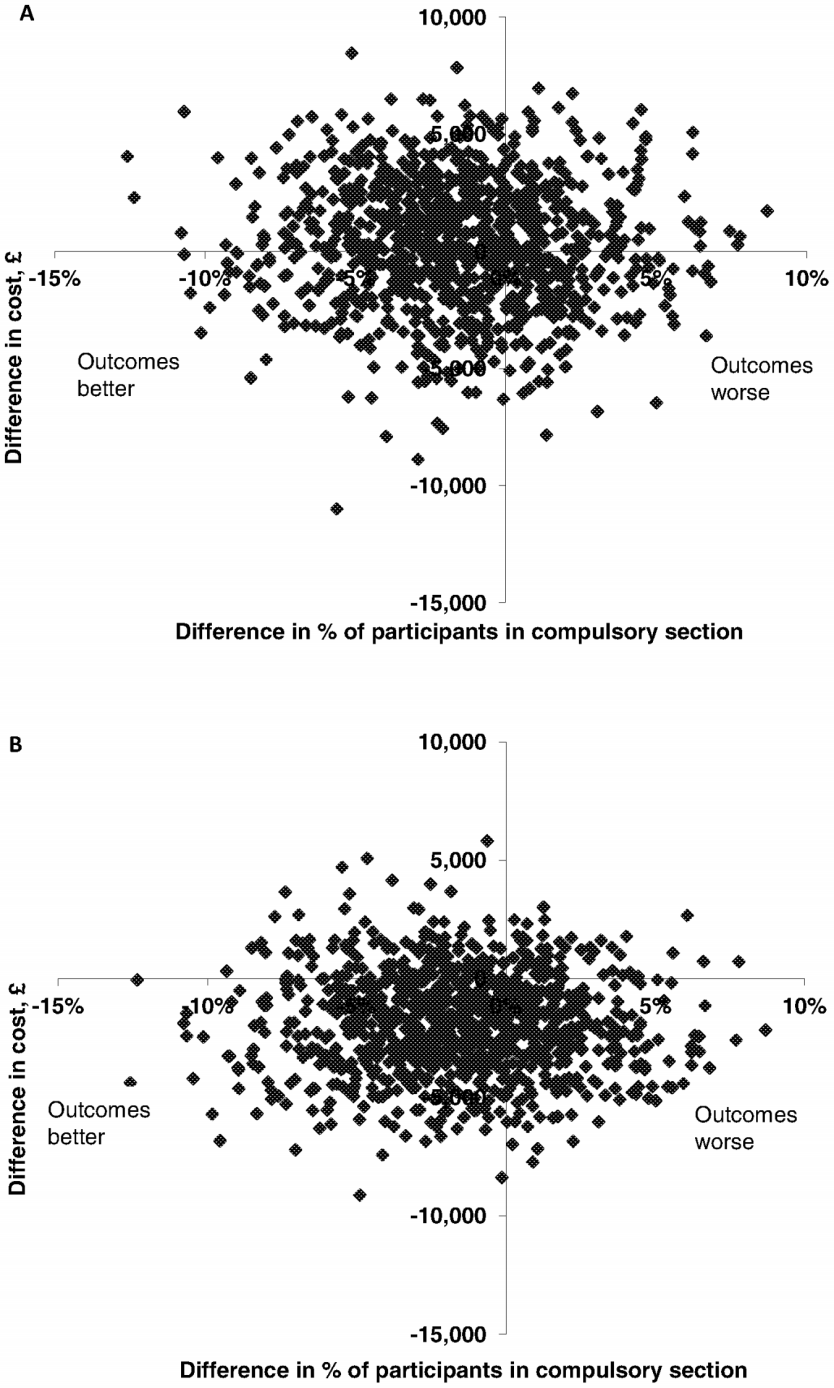
Cost-effectiveness plane. A) Showing bootstrapped cost and effectiveness pairs for societal costs, and B) showing bootstrapped cost and effectiveness pairs for service costs.

Figure 2a shows the scatter plot of the bootstrapped societal cost and effectiveness pairs for JCP versus controls. A larger proportion of the scatter points fall to the left of the y-axis on the cost-effectiveness plane, indicating that the JCP group was associated with greater levels of effectiveness than the control group (smaller proportion of participants subject to a compulsory admission), and were evenly distributed above and below the x-axis, indicating similar levels of societal costs. The scatter plot in Figure 2b shows the bootstrapped replications for service costs which fall mainly in the South-West quadrant, indicating that the JCP group was associated with greater effectiveness (left of the y-axis) and lower cost (below the x-axis).

**Figure 3:**
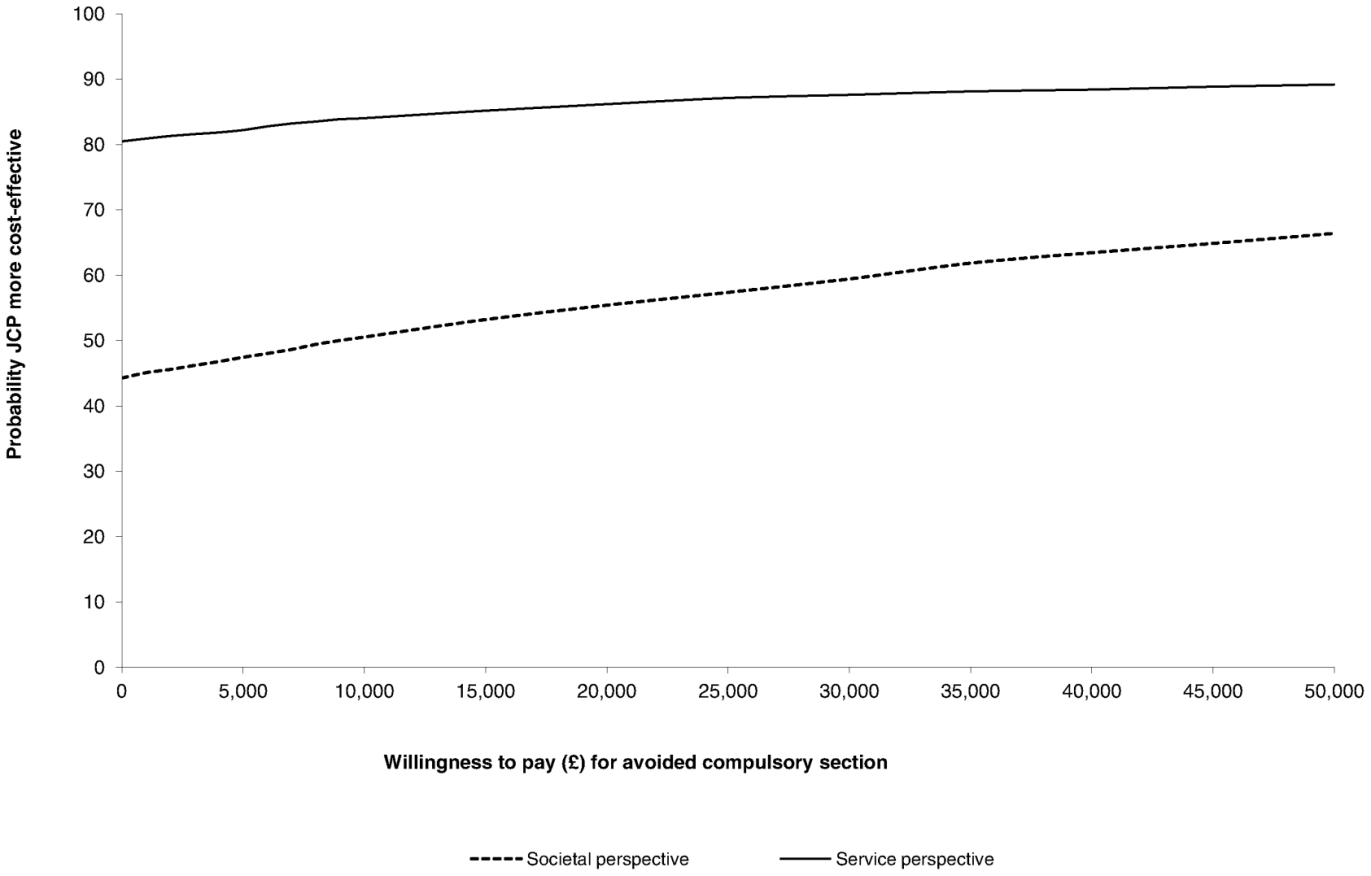
Cost-effectiveness acceptability curve showing probability that JCP+TAU is more cost-effective than TAU over 18-months follow-up.

The cost-effectiveness acceptability curve in Figure 3 shows that from a societal perspective, if society is unwilling to spend any additional amount to reduce compulsory admissions (willingness to pay is zero), then the probability of the JCP being the more cost-effective option is 44%, and thus the probability of the control treatment being the more cost-effective option is 66%. However, the probability of the JCP being the more cost-effective option increases as willingness to pay increases, becoming 50% or more at willingness to pay levels of £9,000 and above. From a service cost perspective the picture is clearer, with the probability of the JCP being the more cost-effective option being at least 80% for every value of willingness to pay.

### Cost and cost-effectiveness by ethnic group

Table 3 reports the main cost and effect differences between the JCP and control group by ethnic group. Results varied noticeably between ethnic groups. For the White group, costs were higher, on average, for the JCP than the control group and there was no difference in effects. For the Black group, costs were lower for the JCP group and effects were better. In the Asian group, costs were higher for the JCP group and effects were worse.

**Table 3:** Costs and outcomes by ethnic group.

	JCP group		TAU group					
	N	Mean (SD)	N	Mean (SD)	Mean difference/odds ratio	95% CI*	p-value*	Summary JCP compared to control
White (n = 314)								
Societal cost# (£)	147	22469 (27611)	167	19823 (32882)	2646	−2987 to 9429	0·337	Costs higher, No difference in outcomes
Service cost∧(£)	147	17680 (20505)	167	16013 (24435)	1667	−3221 to 6360	0·386	
Compulsory admissions, n (%)	164	26 (16%)	178	28 (16%)	0.952	0.532 to 1.706	0·166	
Black/Black British (n = 129)								
Societal cost# (£)	60	23150 (29588)	69	32780 (41170)	−9630	−21043 to 3106	0·160	Costs lower, Outcomes better
Service cost∧(£)	60	17628 (25163)	69	28377 (36627)	−10749	−20387 to 536	0·079	
Compulsory admissions, n (%)	66	13 (20%)	72	23 (32%)	0.553	0.249 to 1.226	0·256	
Asian/Asian British (n = 51)								
Societal cost# (£)	29	22779 (29672)	22	12784 (16444)	9995	−2115 to 24821	0·135	Costs higher, Outcomes worse
Service cost∧ (£)	29	14536 (14384)	22	12018 (16761)	2518	−5267 to 12137	0·853	
Compulsory admissions, n (%)	32	9 (27%)	24	3 (14%)	7.538	0.867 to 65.520	0·139	

* Analysis of cost data adjusted for baseline costs and study site; analysis of compulsory admissions adjusted for study site.

# Total service costs include JCP intervention, health and social care, accommodation and criminal justice services.

∧ Total societal costs include all service costs plus the costs of crimes and lost employment.

**Figure 4:**
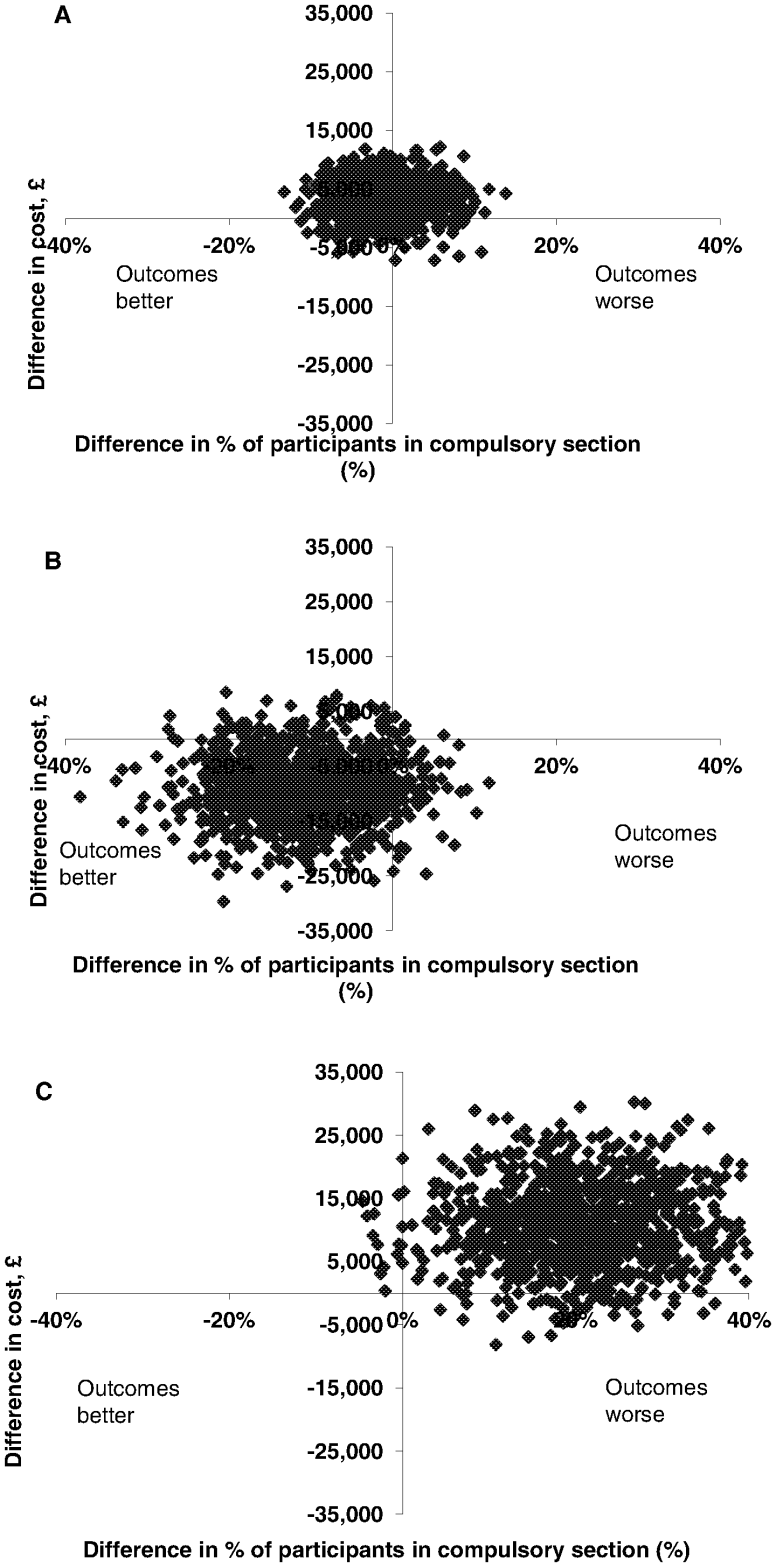
Cost-effectiveness plane. A) Showing bootstrapped cost and effectiveness pairs for Whites, using societal costs. B) Showing bootstrapped cost and effectiveness pairs for Blacks, using societal costs. C) Showing bootstrapped cost and effectiveness pairs for Asians, using societal costs.

**Figure 5:**
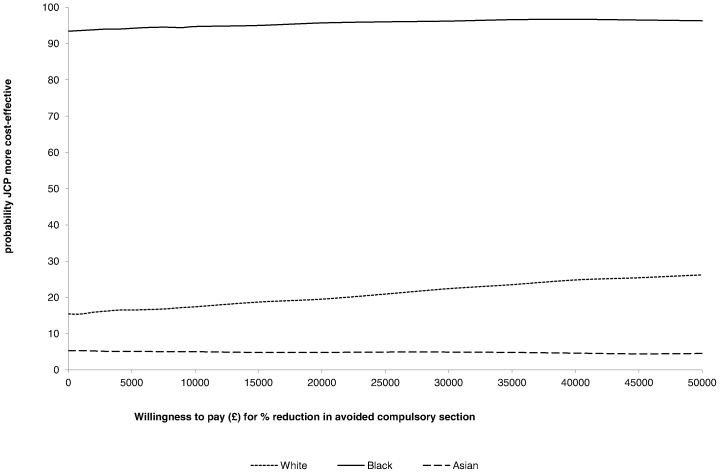
Cost-effectiveness acceptability curve showing probability that JCP+TAU is more cost-effective than TAU over 18-months follow-up by ethnic group.

The scatter plots of bootstrapped replications by ethnic group, using societal costs for a more conservative approach, are shown in Figures 4a to 4c and clearly illustrate this variation. The bootstrapped replications for the Black ethnic group (Figure 4b) fall mainly in the South-West quadrant, showing the JCP group dominated the controls (better outcomes and lower costs). The opposite is true for the Asian ethnic group (Figure 4c) with the scatter falling mainly in the North-East quadrant (poorer outcomes and higher costs). For the White ethnic group, replications fall mainly within the North quadrants (Figure 4a), demonstrating little difference in effects alongside higher costs. The cost-effectiveness acceptability curves for each ethnic group, shown in Figure 5, illustrate that there was at least a 90% probability of the JCP intervention being more cost-effective than the control condition for the Black ethnic group, but less than a 30% chance for the White group and less than a 10% chance for the Asian group.

Finally, the analyses explored the impact of missing data using multiple imputation, increasing the sample size from the 504 with full data to the full recruited sample of 569. None of these sensitivity analyses altered the main findings of the base-case analysis, with costs remaining similar and non-significantly different between the two groups (Table 4).

**Table 4:** Summary of costs and sensitivity analyses.

	N	JCP group Mean (SD)	TAU group Mean (SD)	Mean difference	95% CI	p-value
Service costs#	504	17,223 (21,013)	19,217 (28,133)	−1,994	−5,733 to 2,248	0·414
Societal costs∧	504	22,501 (28,103)	22,851 (34,532)	−350	−4,727 to 5,404	0·902
Productivity losses zero	504	22,485 (28,112)	22,757 (34,563)	−272	−4,846 to 5,684	0·878
Lower cost of JCP intervention$	504	22,430 (28,105)	22,851 (34,532)	−421	−1,998 to 5,534	0·922
Missing data included via multiple imputation	569	22,575 (25,930)	22,819 (33,339)	−244	−4,744 to 4,599	0·976

# Total service costs include JCP intervention, health and social care, accommodation and criminal justice services.

∧ Total societal costs include all service costs plus the costs of crimes and lost employment.

$ Lower cost of the JCP intervention to reflect that facilitators would facilitate more JCPs if the programme were rolled out.

## Discussion

### Cost-effectiveness

The addition of JCPs to usual care for patients with a history of relapsing psychotic illness had no significant effect on compulsory admissions, no significant impact on total societal cost per participant over 18-months, and there was no clear evidence to suggest that JCPs were more cost-effective than usual care from a societal perspective. The cost-effectiveness picture is clearer from the narrower service cost perspective, of particular interest to public sector policy-makers, with evidence to suggest the JCP intervention has a high probability of being the more cost-effective option.

### Ethnic group differences

The whole group analysis hides remarkably distinct patterns of costs and effects for the three main ethnic groups in this study. Whilst the evidence presented does not support the cost-effectiveness of JCPs for either White or Asian ethnic groups, the opposite is true for the Black ethnic group. Despite basing the cost-effectiveness analysis by ethnic group on societal costs, the more conservative approach, the JCP group dominated usual care in the Black ethnic group, with cost-effectiveness analysis suggesting at least a 90% probability of the JCP intervention being more cost-effective than the control condition.

This is an intriguing and possibly important finding. It has been known for several decades that rates of compulsory admission are substantially higher for psychiatric patients from Black ethnic groups compared with other ethnic groups [26]–[28]. It is also clear that service satisfaction is lower among Black psychiatric patients [29]. This generates the hypotheses that trust may be lower [30] and anticipated discrimination higher [31] in Black patients than in other ethnic groups, with JCPs perhaps being associated with greater experience of feeling respected and understood by clinicians in this sub-group than usual services.

### Limitations

The results are limited by the sample size of the study and the possibility that these samples were inadequate to demonstrate statistically significant differences in costs. Commonly, the absence of statistically significant differences in costs (demonstrated here) and in effects (demonstrated in the accompanying paper [4]), would result in the rejection of the hypothesis that a new intervention was more cost-effective than usual care and the continued funding of usual care. More recently, however, the appropriateness of such conclusions has been questioned [10], [11]. Although observed differences may indeed be the result of chance, a decision still has to be made and it is argued that it is better to make the most of the data available and to select the intervention with the highest probability of being cost-effective, at least until further evidence becomes available.

This study is also limited by the lack of a generic, quality of life measure of outcome. Such measures are preferred for economic evaluation because they can be applied to a broad range of patient groups, allowing comparison of cost-effectiveness between interventions for the same disease and between different categories of disease [32]. Generic measures, such as the quality adjusted life year (QALY) preferred by NICE [33], can be associated with societal values of willingness to pay for a unit improvement in outcome that can be used to support resource allocation decisions across an entire health system. However, the JCP intervention was not anticipated to have an impact on health-related quality of life thus such an analysis was considered inappropriate at the design stage.

### Implications

One implication of these results is that specific interventions or service models may be of benefit for groups less well served by mental health services in England. This is supported by the results of two recent pilot studies showing positive results for community mental health teams directly orientated to treating Black people with mental illness [34], [35]. The results reported here are sufficiently striking to warrant further investigation into the potential for patient gain from JCPs specifically among Black patient groups.

## Supporting Information

Protocol S1 **Protocol for the CHAMP trial.**
(DOC)

Checklist S1 **Consort checklist.**
(DOC)
