# Supplementary material for: Randomised Controlled Trial of Joint Crisis Plans to Reduce Compulsory Treatment for People with Psychosis: Economic Outcomes
Source: PLoS One. 2013 Nov 25;8(11):e74210. doi: 10.1371/journal.pone.0074210 (PMC3839936; doi:10.1371/journal.pone.0074210)
Supplement: Protocol S1 — Protocol for the CHAMP trial. (DOC) [file pone.0074210.s001.doc]

**CONTENTS**

[1. General Information 2](#__RefHeading___Toc200359517)

[2. Protocol Amendments and Approvals 5](#__RefHeading___Toc200359518)

[3. Background Information 7](#__RefHeading___Toc200359519)

[4. Trial Objective 7](#__RefHeading___Toc200359520)

[5. Trial Design 8](#__RefHeading___Toc200359521)

[6. The Intervention 9](#__RefHeading___Toc200359522)

[7. Assessments 9](#__RefHeading___Toc200359523)

[8. Statistics 11](#__RefHeading___Toc200359524)

[9. Risks 12](#__RefHeading___Toc200359525)

[10. Trial Supervision 13](#__RefHeading___Toc200359526)

[11. Data Handling And Record Keeping 13](#__RefHeading___Toc200359527)

[12. Data Access 13](#__RefHeading___Toc200359528)

[13. Publication 13](#__RefHeading___Toc200359529)

[14. References 15](#__RefHeading___Toc200359530)

[Appendix 1 – The CRIMSON Study Team 17](#__RefHeading___Toc200359531)

[Appendix 2 – Contact List 18](#__RefHeading___Toc200359532)

[Appendix 3 – Risks to blinding 19](#__RefHeading___Toc200359533)

[Appendix 4 – Analysis Plan. 23](#__RefHeading___Toc200359534)

[Appendix 5 - Publication Protocol 29](#__RefHeading___Toc200359535)

# General Information

| ISRCTN: | 11501328 |
| --- | --- |
| MRC REFERENCE: | G0601660 |
| Trial Office | The CRIMSON office Section of Community Mental Health Box PO29  Health Service and Population Research Department  Institute of Psychiatry  King's College London  De Crespigney Park  London SE5 8AF  Phone: +44 (0)20 7848 5098  [crimson@iop.kcl.ac.uk](mailto:crimson@iop.kcl.ac.uk) |
| Funding | *Medical Research Council (research funding)*  MRC Reference: G0601660  20 Park Crescent  London,  W1B 1AL  Programme Manager: Dr Gavin Malloch |
|  | *Dept of Health* *(excess treatment costs)*  Room 720  Wellington House  133-155 Waterloo Road  London SE1 8UG  Contact: Trudi Simmons  Email: trudi.simmons@dh.gsi.gov.uk  Tel: 020 7972 4895 |
| Sponsor | *Kings College London*  c/o Dr Gill Lambert  Slam/IOP R&D Office  Institute of Psychiatry  De Crespigny Park  Denmark Hill  London  SE5 8AF  Email: [Gill.Lambert@iop.kcl.ac.uk](mailto:Gill.Lambert@iop.kcl.ac.uk) |
| Ethics | *Kings College Hospital Research Ethics*  Main Submission  Reference number: 07/H0808/174  Approval: 10 January 2008  Amendment  Reference: Amendment 1 date 22.4.2008  Approval: 6 May 2008  Amendment  Reference: Amendment 2, September 2008  Approval: 31 October 2008  Amendment  Reference: Amendment 3, October 2009  Approval: 30 November 2009 |

| Collaborating Institutions | *University of Manchester*  *University of Birmingham* | |
| --- | --- | --- |
| Chief Investigator | *Professor Graham Thornicroft*  Section of Community Mental Health Box PO29  Health Service and Population Research Department  Institute of Psychiatry  King's College London  De Crespigney Park  London SE5 8AF | |
| **Principal Investigators** | Manchester/Lancashire: | Professor Max Marshall |
|  |  | Dr Waquas Waheed |
|  | Birmingham | Professor Max Birchwood |
|  | London | Professor George Szmukler |
| **Trial Statisticians** | Dr Morven Leese  Professor Graham Dunn | |
| **Scientific Coordinator** | Ms Simone Farrelly | |
| **Project Team** | See Appendix 1 & 2 | |
| **Advisor** | Professor Dinesh Bhugra  Professor Jeffrey Swanson | |
| **Trial Steering Committee** | Professor Simon Gilbody  Paul Farmer  Peter Campbell  Morven Roberts  Mary Evans  Genevra Richardson | |
| **DMEC** | Tim Croudach  Ian Harvey  Mike Crawford (chair) | |
| **Start date** | 1 October 2007 | |
| **End date** | 31 March 2012 | |

# Protocol Amendments and Approvals

The following changes and approvals have been made to the protocol.

| ***Version Number*** | ***Date*** | ***Submitted/shown to*** | ***Amendments*** | ***Section*** |
| --- | --- | --- | --- | --- |
| 1 | 29 September 2006 | MRC grants office |  |  |
| 2 | 23 October 2007 | n/a | Add assessment of CPA review | Control intervention |
| 3 | 31 October 2007 | Approved by ethics | Focus group timing and overall sample numbers  Revised numbering of references | Proposed trial  Reference |
| 4 | 6 May 2008 | Approved by ethics 06 May 2008 | Add CC socio demogs and JCP experience study | Proposed trial |
| 5 | 4 June 2008 | Ethics committee 23 July 2008 | Reformat protocol for submission to Lancet. Content not changed. Addition of: ‘General information’ section; protocol amendment and approvals section; |  |
| 6 | 22 September 2008 | Ethics committee | - Addition of Service Engagement Scale (SES) - Update study team lists and analysis plan | - 7 – Assessments - Appendices |
| 7 | 21 January 2009 |  | - addition of recruitment projections/plan chart - detail of length of recruitment period - ‘detained or treated’ added to the description of primary outcome - Change of ‘societal’ to ‘broad’ perspective in description of economic analysis - Revised Enhanced CPA criterion following new guidelines | - Appendix 3 - Section 5 - Section 4 - Section 7.3 |
| 8 | 20 April 2009 |  | - clarification of eligibility criteria ‘adults (aged 16 and above)..’ | Eligibility criteria |
| 9 | 23 September 2009 | Ethics committee  30/11/09 | - Trial end date altered post extension from MRC - update TSC membership and DMEC membership - update methodology for CPA audit - addition of psychiatrist interviews to qualitative studies | General Information, appendix 3 and section 10  6. The intervention  7.2. Qualitative studies |
| 10 | 22 February 2011 | Ethics committee 31/3/2011 | - collection of demographic data of refusals - contacting refusers of intervention to clarify further involvement | 5.1 and 7.1 |
| **11** | **24 May 2011** | **Awaiting approval** | **- adding individual interviews for sectioned intervention participants.** | **7.2** |
|  |  |  |  |  |
|  |  |  |  |  |
|  |  |  |  |  |
|  |  |  |  |  |
|  |  |  |  |  |

# Background Information

Two of the key guiding principles of mental health policy in England are: (i) that service users should exercise choice and control over their treatment^1^, and (ii) be subjected to the least restrictive form of care. Service users now routinely participate in care planning, service development and research. Nevertheless, against the European trend^2^, the use of compulsory treatment in England has continued to rise, with a 15% increase in compulsory detention under the Mental Health Act (MHA) from 1993 to 2003. This conflicts with government policy and service user preferences, and is also a concern given that many service users find in-patient wards untherapeutic^3^. The Joint Crisis Plan is so far the only structured intervention which has been shown to reduce compulsory treatment.

A Joint Crisis Plan (JCP) aims to empower the holder and to facilitate early detection and treatment of relapse^4^. It is developed by a mental health service user in collaboration with staff. Held by the service user, it contains his or her treatment preferences for any future psychiatric emergency, when he or she may be too unwell to express clear views. The JCP format has developed over the last decade after widespread consultation with national service user groups, interviews with organisations and individuals using JCPs^5^, and after detailed developmental work with service users in South London. Initial findings from the pilot study suggest that the negotiation process itself helps engage the service user and the clinician in a more acceptable, collaborative, and beneficial form of therapeutic relationship^6^. JCPs are somewhat similar to Psychiatric Advance Directives (PAD), but also differ as JCPs do not have any degree of medico-legal enforceability. Studies in the USA have found that PADs produce high levels of demand and satisfaction^7-9^.

In England there is particular concern that black service users experience more coercive mental health care than their white counterparts^10-14^. Specifically, higher prevalence rates of mental illness have been found among black Caribbeans in the UK, and higher admission rates for black Caribbean and black African than white service users have been found for compulsory admission under the MHA, and use of intensive care, medium-security and high-security psychiatric units^10;15-30^. So far no interventions have been identified which clearly reduce compulsory mental heath treatment for black Caribbean and black African service users.

A pilot study of JCPs conducted by the applicants in South London^33^ showed that at 6-12 month follow-up, 57% of participating psychiatric service users with JCPs reported feeling more involved in their care, 60% were positive about their situation, 51% felt more in control of their mental health problem and 41% felt they were more likely to continue treatment. Further, a recently completed exploratory randomised study of JCPs^34^, found that use of the MHA was significantly reduced for the intervention group, 10/80 (12.5%) of whom experienced compulsion versus 21/80 (26.5%) of the control group (risk ratio 0.48, 95% CI 0.24 to 0.95, p = 0.028). While overall bed-day use was not significantly different between experimental and control groups, the mean number of days detained for the intervention group during the 15 month follow-up period was 14 compared to 31 for the control group (difference 16, 95% CI 0 to 36, p=0.04). We concluded that coercive treatment in the form of MHA use was halved by the use of JCPs: the first structured clinical intervention shown to reduce compulsion in mental health services. JCPs are therefore relatively straightforward to implement, and offer the prospect of less restrictive care for service users with psychotic disorders. A more recent health economic analysis of this trial has shown that the intervention is highly likely to be cost-effective^35^. The time is therefore right for a definitive trial^36^.

# Trial Objective

***Primary hypothesis***

(i) JCPs will significantly reduce the proportion of service users detained or treated under a section of the Mental Health Act at any point during the follow-up period, compared with the control condition.

***Secondary hypotheses***

Compared with the control condition, JCP use will result in significant improvements in:

(ii) total costs over the follow-up period and so relative cost-effectiveness

(iii) perceived coercion

1. service user engagement with mental health services
2. therapeutic alliance
3. the use of the Mental Health Act for the Black service users during follow up.

**Therapeutic processes**

We shall also undertake exploratory assessments and analyses of whether (i) service user trust; and (ii) ‘sealing’ (the recovery style of each service user)^31^ are affected by JCPs. Sealing is a form of 'denial' and is motivated by individual vulnerability in coping with life events. As the basis for JCPs is the development of a collaborative, trusting relationship, such a non-confrontational style is expected to protect against sealing and hence disengagement from services. We expect that particular variables may have explanatory power as mechanisms of action of JCPs, such as shared decision making^32^, but without prior evidence of this we cannot set formal hypotheses at this stage.

# Trial Design

This is a 3 centre definitive randomised controlled trial to establish if Joint Crisis Plans (JCPs) are effective, both for all mental health service users, and for those from black (black Caribbean and black African) communities. The hypotheses to be tested are whether, compared with treatment as usual, JCPs improve: the proportion of service users treated under a section of the Mental Health Act, total costs, perceived coercion, service user engagement with mental health services, therapeutic alliance, and use of the Mental Health Act for the Black service users.

This study is an individual-level single-blind RCT of JCPs compared with a treatment as usual control condition for people with a history of relapsing psychotic illness in Birmingham, London and Lancashire/Manchester. Service users under the care of community services in each of the participating inner-city sites will be identified by Care Programme Approach and IT record systems and Care Co-ordinator case lists, and informed consent will be sought to participate in the study. Ethnicity will be self-defined according to usual Office of National Statistics (ONS) procedures. The total duration of the study will be four years, to allow for the recruitment to target numbers of subjects over 18 months, standardised training, provision of the manualised intervention, follow-up assessments, and data analysis, using intention to treat methods.

**5.1 Randomisation procedure and methods to minimise bias**

A list, generated by Clinical Studies Officers from the Mental Health Research Network, will be maintained by the Study Co-ordinating Centre of all service users at each local site fulfilling the inclusion and exclusion criteria for the study. Each eligible person will then be approached by a member of the research team and invited to participate in the study, thus eliminating selection biases. A randomisation list of those eligible and consenting, stratified by centre, will be prepared using randomised permuted blocks of randomly varying block size, with equal allocation to the two arms. The randomisation will be managed by an independent system that will provide random allocation to all sites when provided with subject identification number, and so ensure the concealment of allocation of service user status in the two arms of the trial. Confirmation of eligibility and consent, and minimal baseline data will be obtained prior to randomisation.

The co-ordinating centre will scrutinise the implementation of the JCP intervention across sites to ensure fidelity and comparability of the intervention are preserved, using a rating scale for this purpose along with analysis of taped interviews.

Bias in the recruitment and randomisation process will be avoided by having randomisation done centrally by an independent system, and hence concealment maintained from the investigators, and by keeping a log of all service users randomised and including them all in the analysis. Research workers, who will collect outcome data, will be blinded to the allocation. Some outcome data (in particular the primary outcome) will be obtained from staff, case notes, and administrative sources, even for service users lost to follow-up interview. Bias in the outcome data will also be minimised by the use of standardised objective assessments and by blinding the researchers as far as possible to service user allocation. In addition there will be specific instructions to participants and clinical teams not to disclose treatment details; along with separate administrative functions and office space with clear protocols and protected data storage in line with Good Clinical Practice Guidelines (full blinding procedures available in Appendix 3). As is common in effectiveness trials^46^, every effort will be made to maximise the single blindness of research raters, whose ‘best guesses’ of service user status will be assessed to test whether their blindness is maintained*.*

Likewise, demographic information of trial refusers will be collected to accurately assess the representativeness of the final trial sample and to assess whether there were any biases in the recruitment of trial participants..

**5.2 Inclusion criteria**

Eligible service users be adults (age 16+) and will have: (i) contact with a local Community Mental Health Team (CMHT) (will include assertive outreach teams, early intervention teams, and community forensic teams, but not home treatment teams.); (ii) have been admitted to a psychiatric in-patient service at least once in the previous two years; (iii) have a diagnosis of psychotic illness, including bipolar affective disorder (using Operational Criteria Checklist OPCRIT ^47^), and (iv) have been on the local NHS Trust Enhanced CPA/CPA Register in the last two years. We shall include service users who do not speak English. For non-English speakers, both written translation and interpreters are needed and we shall employ interpreters as required.

**5.3 Exclusion criteria**

**Exclusion criteria.** Those unable to give informed consent. Current in-patients will not be recruited to avoid any perceived potential coercion to participate, nor any patient subject to a compulsory community treatment order. No other exclusions will be made, to maximise the external validity of the trial.

# The Intervention

**6.1 Experimental Intervention:** At each site, a trained and clinically experienced Facilitator will organise a meeting with each service user randomised to receive a JCP. The Facilitator will introduce the JCP ‘menu’ (a list of topics to be considered for inclusion in the JCP) to each service user. He/she will then organise a meeting between the service user, team consultant psychiatrists or other psychiatrist, and the Care Co-*ordinator/ treating doctor’,* when the JCP contents will be finalised. The service user is encouraged to bring a carer or friend to act as an advocate. The JCP contains: information on early warning signs of relapse and advance treatment statements; contact details of primary and secondary care staff for routine and emergency care; details of medication; psychiatric and physical diagnoses; allergies; and details of who will have a copy of the JCP. The Facilitator produces a typed version of the JCP, computer-generated to allow replacement and updating, and it will also be included on computerised patient information systems where these are in routine use by the services. Copies will be sent to all those whom the service user specifies.

**6.2 Control intervention.** After careful consideration, we have chosen to use a treatment as usual (TAU) control condition, as this provides a fair comparison with routine clinical practice, and will answer the question of whether JCP use is superior to current standard care. TAU includes, as a part of the Care Programme Approach (CPA), the need for service users to receive written copies of their care plan^3^, including a ‘crisis contingency plan’, as indeed was the case in our recent exploratory trial. We expect that the CPA arrangements in secondary care, and the new 2006 Quality Outcome Framework (QOF) to have a care plan documented in primary care case records, will be applied equally by routine services to intervention and control groups, and there is little evidence for differential QOF implementation between practices. We shall assess the content of the Care Programme Approach of all participants at baseline and follow-up.

**6.3. Training and Fidelity of Intervention**

Full details of the intervention have been published elsewhere and the intervention was manualised in the exploratory trial^44^;. Facilitators will be experienced mental health workers who will receive 5 day training in the process of negotiating joint crisis planning meetings, and producing JCPs. Continuing supervision will be provided during the study period. Fidelity to model will be assessed following training and at random points during the study using a rating scale to evaluate audiotapes of meetings and copies of completed JCPs. A manual is available and a full training video will be produced incorporating training material from the trial.

# Assessments

**7.1. Primary outcome.**

**Primary:**

1. Proportion of service users admitted or otherwise subsequently detained under an order of the Mental Health Act during the follow-up period. MHA data will be gathered from and validated between the following sources: case notes, the local Patient Administration System, Mental Health Act Office data, and interviews of service users and Care Co-ordinators.

**Secondary:**

1. Cost as measured by the Adult Service-Use Schedule (see section 3.11)
2. Perceived coercion (Admission Experience Inventory)^48^
3. Engagement with mental health services (Engagement and Acceptance Scale^49^ and Service Engagement Scale ^60^
4. Therapeutic relationship for service users and staff (Working Alliance Inventory)^50^

In addition socio-demographic data will be collected at baseline, and social and functional clinical descriptors (baseline and follow-up) will be gathered along with the Global Assessment of Function^51^. We shall also record: involuntary transport to hospital under the MHA, police involvement in admission, incidents of violence, incidents of self harm, and criminal justice system involvement. In addition we shall conduct systematic analyses of the content of these JCPs in terms of treatment preferences, instructions, information, and consent or refusal of interventions. Ethnicity will be described using ONS categories as they have been thoroughly tested and are known to be acceptable to the majority of the population^52^. Additionally, socio-demographic data and length of practice data will be collected for care co-ordinators.

To ensure the accurate reporting of the representativeness of the trial sample, we will collect basic demographic data (age, gender, diagnosis, ethnicity and last hospital admission) on those who refused to participate in the trial.

At follow-up we will contact all participants who originally participated in the trial. Intervention participants who refused to complete a Joint Crisis Plan, will be contacted via a letter to clarify their wishes for any further involvement in the trial.

#### 7.2 Qualitative evaluation

Qualitative studies will be carried out to explore the processes through which JCPs achieve change in practice. Pilot work suggests they have direct and indirect effects. Negotiating JCP content may clarify treatment issues and build consensus between service users and staff. However effects on trust (based upon the approach of Mechanic)^53^, service user engagement in the process of care including shared decision making^32^, changes to service user self esteem, and empowerment, clearer channels of communication between all parties, changes to staff risk perception and/or changes within the culture of the mental health service may also be important. Focus groups, a potentially more enabling setting for vulnerable users to express their views than individual interviews, will be used to examine people’s experience of the JCP. The dynamic interaction of the group can also provide insights into attitudes, perceptions and opinions and dissent between participants can clarify beliefs and reveal underlying assumptions. The important ‘break characteristic’ used in data analysis will be whether service users with a JCP were subject to coercion (MHA use). 18 focus groups (6 groups in each site) will run 18-21 months after entry to the study.

Separate service user and health professional focus groups with service users who have or have not been coerced as defined will be followed, usually one week later, by a combined subset of the service users and health professionals who attended the separate groups. We expect to involve 16 service users in each geographical site and a similar number of professionals involved in the development of the JCP with the service user. This tripartite technique has been used with good effect by one of the applicants^59^. Focus group topic guides will be developed from a literature review and include the themes discussed above^54-56^. Combined groups will additionally explore the roles and responsibilities of service users and health care professionals in relation to the use of the JCP and the influence on and impact of the culture of mental health services. **If there are insufficient numbers to arrange focus groups in any of the sites, individual interviews will be conducted to collect detailed information of individuals’ experiences of the intervention.**

To understand the experiences and views of psychiatrists who have participated in the intervention, a series of individual interviews will be conducted. Psychiatrists, being ultimately responsible for the service user’s care are a key stakeholder in such an intervention; their engagement in the process is potentially key to its success. Interviews will cover the psychiatrist’s views on the process of the intervention, the outcome and the impact of such an intervention on mental health services. Interviews will be conducted in all sites, and will continue until ‘saturation’ is achieved i.e., no further themes are identified.

Focus groups **and individual interviews (with ~~and~~ psychiatrists and others) ~~interviews~~** will be audio taped and fully transcribed. Transcripts and notes will be read and re-read independently by two of the research team. The data will then be organised into initial codes and higher codes that provide insight into emergent themes. For the focus groups, reliability will be enhanced by identifying issues that are consistent between groups and validated using so-called ‘sensitive moments’ within group interactions that indicate difficult but important issues. A computer software package will be used to manage the data and increase the transparency of the analysis. Deviant cases will be actively sought throughout the analysis and emerging ideas and themes modified in response.

To gain further insight into the experience of developing and holding a Joint Crisis Plan, we will invite two participants per site to partake in further interviews. Interviews will explore the experiences of intervention group participants during the 18 month follow-up period, including their views on JCP development, and use of and adherence to the JCP in the event of a relapse. These interviews will be conducted by researchers after JCP development, and every five months during the study period. We will be sensitive to the circumstances surrounding these interviews to avoid any sense of coercion or contributing to participant stress levels. The service users participating in these interviews will not be invited to take part in focus groups. These interviews will be digitally filmed and/or audio-taped depending on participant preferences.

**Outcome measures**

| **Therapeutic Process** | **Standardised measure** | **Source** | **Time** |
| --- | --- | --- | --- |
| Trust | Mechanic Trust Schedule^57^ | Focus Groups | FU |
| Recovery Style | Recovery Style Q’re ^58,59^ | Self-report | B FU |

| **Outcome** | **Standardised measure** | **Source** | **Time** |
| --- | --- | --- | --- |
| MHA use | Routine hospital datasets | Admin. records | B FU |
| Costs | Adult Service Use Schedule | Interview | B FU |
| Perceived coercion | Admission Experience Inventory (Community)^48^ | Self report | B FU |
| Engagement with care | Engagement and Acceptance Scale ^49^ and **Service Engagement Scale ^60^** | Staff self report | B FU |
| Therapeutic relationship | Working Alliance Inventory^50^ | Self-report | B FU |

B=Baseline, FU Follow-up at 18 months post baseline

**7.3 Economic Evaluation**

An economic evaluation will be included to explore the relative cost-effectiveness of the JCP compared with the control condition in terms of the primary outcome measure (proportion of service users under an order of the Mental Health Act at any point during the follow-up period). For ease of comparison across different interventions, current convention recommends reporting cost-effectiveness in terms of health-related quality adjusted life years (QALY). However, the JCP intervention is not anticipated to have an impact on health-related quality of life thus such an analysis would be inappropriate.

The economic evaluation will take a broad perspective and will be based on comprehensive resource use data collected on all health, social care, housing and other community support services used by individual trial participants, contact with criminal justice agencies, and other resources arising from the use of the MHA, and productivity losses. Data will be collected in interview with participants at baseline and follow-up using the Adult Service Use Schedule (AD-SUS). Interview data will be supplemented by data on hospital contacts, the key cost driver in this population, collected from routine computerised hospital records. Data on the professionals and time involved in setting up the JCP intervention will be collected from JCP facilitator records, as in our previous trial^64^.

# Statistics

**8.1. Power calculation**

The primary outcome is reduction in the proportion of service users admitted or detained under a Mental Health Act section at least once, obtained from records. In the pilot trial based in London 26% were compulsorily admitted over 15 months, equivalent to 30% over 18 months. Routine data for inner city wards in Birmingham and Manchester show a very similar proportion, on average. Assuming that a clinically important reduction would be to at least halve the proportion, i.e. a reduction in absolute terms by 15% to 15%, 90% power using a double-sided test with alpha=0.05 would require 174 in each arm. For the ethnic subgroup analysis (see 3.17), where the baseline compulsory admission rate is likely to be higher, an achieved subsample of 91 per arm would give 80% power to detect a difference from 40% to 20% Given the percentages of service users likely to be black at each site (from recent actual MHA use data), 90 are likely to be found with a sample of 270 per arm, and the minimum achieved would be about 80 with a slight reduction in power. Loss to follow-up is likely to be about 15% for the interview data so this sample size (270) would reduce to an effective 229 per arm, which would allow standardised effect sizes of 0.3 for the secondary outcomes to be detectable with 90% power. The total to be recruited would therefore be 540, or 180 per site (which includes 48 who will also have qualitative assessments after 18 month follow-up).

**8.2. Analysis plan**

The principal analysis of effectiveness will compare the primary and secondary outcome measures at 18 months, combined over centres. The proportions admitted to hospital under a section at follow up will be compared between randomisation groups using logistic regression controlling for centre. Other (continuous) outcomes such as therapeutic alliance and engagement with mental health services will be analysed using analysis of covariance controlling for baseline (pre-intervention) measures and centre. Number of admissions will be analysed using Poisson regression, and time to first admission using survival analysis. Bed-days and other very skewed data will be analysed using bootstrapping to obtain confidence intervals and p-values^70^. Short scales will be analysed using ordered logistic regression. An intention-to-treat analysis will be applied in the first instance (i.e. analysing all available data from service users as randomised). Time trends in measures available at baseline and two time points will be analysed using methods for longitudinal data such as random effects regression. Sensitivity analyses will be performed to assess the influence of loss to follow-up and refusals, including imputation of missing baseline values from the within-centre means; multiple imputation of follow up values (where feasible from other variables) and CACE analyses^65^. (Full analysis plan is in Appendix 4)

**8.3. Health Economic analysis**

The JCP intervention will be costed using recognised micro-costing methods^65^. Costs will be based on the mid-point of the salary scale of the relevant JCP professionals, including all employer costs (National Insurance and Superannuation contributions) and appropriate overhead costs (capital, administration, managerial etc). Other services will be costed using locally applicable estimates of long-run marginal opportunity costs. Where necessary, these will be supplemented by published national unit costs^66^. Nationally applicable unit costs will be applied in sensitivity analysis to assess the generalisability of the cost results to the UK as a whole. Productivity losses will be costed using the human capital approach, which involves multiplying days off work due to illness by the individual’s salary level^67^. A number of commentators have argued that this approach is limited since it tends to overestimate productivity losses by ignoring, for example, the ability to replace workers from the pool of unemployed people. Given these limitations, the impact of varying productivity losses will be explored in sensitivity analysis*.*

The mean costs in the two groups will be compared with confidence intervals for mean differences estimated using bootstrapping. Regression models will be used to adjust for baseline characteristics. Future costs will not be discounted in the main analyses because the single follow-up point will not allow costs that fall within the first 12 months to be separated from those which occur subsequently. However, the potential impact of excluding discounting will be explored in sensitivity analyses. Cost-effectiveness will be explored using the proportion admitted under a section over the 18-month follow-up period. Incremental cost-effectiveness ratios (of the additional costs to the additional effects of the JCP process in comparison to the control group) will be reported^71^ and cost-effectiveness acceptability curves showing the probability that the JCP is more cost-effective than the control condition will be plotted^70^. Supplementary evaluation will take the form of a cost-consequences analysis examining total costs in relation to all other secondary outcomes^69^. SPSS version 12 and Stata version 9 will be used for the analyses.

# Risks

Developing JCPs involves reviewing the service user’s experiences of previous admissions and considering the possibility of future relapses. Some service users can find this stressful, either because they recall distressing experiences or because they develop further insight into the severity of their problems. One potential risk is that the stress of developing a joint crisis plan could be a factor in precipitating further relapse. In the pilot study^43^ this was observed in 1 of 40 service users, who did not need admission. In the earlier RCT^44^ the intervention group had fewer admissions and adverse events (self harm or violence), indicating that the overall effect was to reduce rather than increase risk.

A further potential risk is that service users will make statements refusing essential or life saving treatment, or state a preference for inappropriate treatment. This is a risk present in the development of advance directives and crisis cards, although there is no legal obligation on doctors to act on advance consent to treatment. If the service user and the team are unable to agree that a treatment choice is clinically acceptable the user can choose whether a statement to that effect is included on the Joint Crisis Plan, or the plan will be reclassified as a Crisis Card indicating the lack of agreement. This occurred on only 1 occasion in the pilot study. If an advance refusal of treatment stated on a JCP is considered by staff to put a service user at risk during a crisis the refusal could be over-ruled by the use of the Mental Health Act 1983.

# Trial Supervision

**10.1. Trial Steering Committee**

TSC members will include: Independent members: Chair: Dr Simon Gilbody, Professor of Psychological medicine and Health Services Research, Department of Health Sciences, University of York; Paul Farmer, Chief Executive of MIND, Peter Campbell, Service user and former secretary of Survivors Peak Out; Mary Evans; Genevra Richardson, Centre of Medical Law & Ethics / KCL; and Morven Roberts, Programme Manager, MRC. In addition the Chief Investigator (Graham Thornicroft) the site lead/PIs (George Szmukler, Max Birchwood, Max Marshall, Waquas Waheed) and the Scientific Coordinator will be members of the TSC.

**10.2. Data monitoring and Ethics committee**

Dr Mike Crawford (chair); Dr Tim Croudach; Dr Ian Harvey

**10.3. Project management group**

Project Management Group will meet every two months. Members: Max Birchwood, Lead for the Birmingham trial site; Sarah Byford, Lead for health economic aspects of the trial; Graham Dunn, Co-lead for statistical design and analyses; Dr. Waquas Waheed from the Lancashire Care NHS Trust and Richard Gater, Co-lead for the Manchester trial site; Claire Henderson, Lead for the fidelity and reliability aspects of the trial; Martin Knapp, Lead for the health policy aspects of the trial; Morven Leese, Co-lead for statistical design and analyses; Helen Lester, Lead for focus groups aspect of the trial; Max Marshall, Co-lead for the Manchester trial site; Diana Rose, Lead for trust and therapeutic engagement aspects aspect of the trial and for service user participation; Kim Sutherby, Lead for JCP Facilitator training; George Szmukler, Lead for the London trial site; Graham Thornicroft, Lead applicant, overall responsibility for trial conduct and completion and Simone Farrelly, Scientific Coordinator.

# Data Handling And Record Keeping

All data will be anonymised and entered on a secure database developed and maintained by the Clinical Trials unit at the Institute of Psychiatry. The only documentation which will contain identifying material are the participant contact details, participant invitation letters (containing name and address), consent form (containing name). All paper forms of this data will be stored in locked filing cabinets at each of the sites and transferred to the Study Coordinating Centre at the end of the study. Only the research team will have access to these filing cabinets. All other data will be identified by a Subject Identification Number only. A file linking the Subject Identification number and personal data will be password protected and stored on a secure server at the Study Coordinating Centre. Only the research team will have access to this data.

All members of the study team will receive Good Clinical Practice training and specific instruction regarding

maintaining confidentiality of data.

# Data Access

This study will generate qualitative data comprising interview transcripts and associated analyses, and quantitative data from questionnaires. Exclusive use for primary research by the research team is envisaged for no more than 2 years following the study, to meet our dissemination goals. Both the quantitative and qualitative data will be shared in anonymised form. Secondary re-analysis of data are anticipated as well as contribution to larger datasets of routinely collected outcome data. Archiving and curating (including data sharing agreements and management of access rights) will be undertaken within the framework used by King’s College London, with due attention to issues of ethical (including consent and confidentiality aspects), legal and institutional regulatory permissions. Intellectual property rights for the data will be retained by the lead applicant and King’s College London.

# Publication

The results of the research will be targeted for publication in peer-reviewed journals of general and special interest. Publication protocol is attached in Appendix 4. At the conclusion of the trial, participants will be sent a report outlining the major findings of the study.

# References

(1) Department of Health. London: Department of Health; 2000.

(2) Salize HJ et al, British Journal of Psychiatry 2004; 184:163-168.

(3) Rose D. London: The Sainsbury Centre; 2001.

(4) Sutherby K et al, Acta Psychiatrica Scandinavica 1999; 100 (1):56-61.

(5) Sutherby K et al. Psychiatric Bulletin 1998;(22):4-7.

(6) Martin DJ et al Consulting and Clincal Psychology 2000; 68 (3):438-450.

(7) Backlar P et al Administration and Policy in Mental Health 2001; 28 (6):427-441.

(8) Swanson JW et al Psychiatry 2000; 63 (2):160-172.

(9) Swanson JW, Swartz M, Ferron J, et al. Journal of American Academy Psychiatry and the Law, 2006; 34:43–57

(10) Department of Health. London: Department of Health; 2005.

(11) McKenzie K. British Medical Journal 2003; 326 (7380):65-66.

(12) Morgan C et al Psychological Medicine 2006; 36 (2):239-247.

(13) Morgan C et al British Journal of Psychiatry 2005; 186:290-296.

(14) Morgan C et al British Journal of Psychiatry 2005; 186:281-289.

(15) King M et al British Medical Journal 1994; 309 (6962):1115-1119.

(16) Harrison G et al British Journal of Psychiatry 1999; 175:43-49.

(17) Bebbington P et al Social Psychiatry and Psychiatric Epidemiology 2000; 35 (5):191-197.

(18) Weich S et al Psychological Medicine 2004; 34 (8):1543-1551.

(19) Coid J et al British Journal of Psychiatry 2000; 177:241-247.

(20) Coid J et al British Journal of Psychiatry 2002; 181:473-480.

(21) Coid J et al British Journal of Psychiatry 2002; 181:481-487.

(22) Bhui K. British Journal of Psychiatry 2001; 178:575.

(23) Lelliott P et al British Journal of Psychiatry 2001; 178 (1):62-66.

(24) Harrison G et al British Medical Journal 1996; 312 124.

(25) Davies S et al. Britsh Medical Journal 1996; 312 (7030):533-537

(26) Maden A et al British Journal of Psychiatry 1999; 175:317-321.

(27) Bhui K et al British Journal of Psychiatry 2003; 182:105-116.

(28) Dein K et al Advances in Psychiatric Treatment 2007; 13 (5): 350-357

(29) Department of Health. London: Department of Health; 2005.

(30) Health Commission. London: Health Commission; 2005.

(31) Tait L et al British Journal of Psychiatry 2003; 182:123-128.

(32) Hamann J et al Acta Psychiatrica Scandanavica 2003; 107 (6):403-409.

(33) Sutherby K et al Acta Psychiatrica Scandanavica 1999; 100 (1):56-61.

(34) Henderson C et al British Medical Journal 2004; 329 (7458):136-138.

(35) Flood C. et al. British Medical Journal 2006; 333: 729

(36) Campbell M et al British Medical Journal 2000; 321 (7262):694-696.

(37) Henderson C et al The Cochrane Database of Systematic Reviews 1999; Issue 3.

(38) Geller JL. Psychiatric Quaterly 2000; 71 (1):1-13.

(39) Hoge SK et al Behavior Science and the Law 1993; 11 (3):281-293.

(40) Hiday VA et al International Journal of Law and Psychiatry 1997; 20 (2):227-241.

(41) Papageorgiou A et al British Journal of Psychiatry 2002; 181:513-519.

(42) Department of Health. London: Department of Health; 1999.

(43) Sutherby K et al British Journal of Psychiatry 1996;68 (3):381.

(44). Henderson C et al British Medical Journal 2004; 329, 136

(45) Gardner W et al Behavior Science and the Law 1993; 11 (3):307-321.

(46) Tansella M et al, Psychological Medicine 2006; 36, 711-720

(47) McGuffin P et al Archives of General Psychiatry 1991; 48:764-770

(48) Gardner W, et al Behavior Science and the Law. 1993; 11:307-321**.**.

(49) Park M. J., et al Psychological Medicine, 2002; 32, 855-861

(50) Busseri MA et al Psychological Assessesment 2003; 15 (2):193-197.

(51) American Psychiatric Association. Washington D.C. American Psychiatric Association; 1987.

(52) Department of Health. London: Department of Health; 2005.

(53). Mechanic D. & Meyer S. Social Science & Medicine 2000; 51, 657-668.

(54) Kitzinger J. Sociology of Health and Illness 1994; 16:103-120.

(55) Lester H et al British Medical Journal 2005; 330:1122-1128.

(56) Barbour R et al London: Sage; 1999

(57) Mechanic D. & Meyer S. Social Science & Medicine, 2000; 51, 657-668.

(58) Tait L. et al British Journal of Psychiatry 2004;, 180 (5) , 410 -415

(59) McGlashan,T.H.,Wadeson,H. S.,Carpenter,W., et al Journal of Nervous and Mental Disease, 1977; 164,182-190

(60) Tait et al Journal of Mental Health, 2002; 11(2), 191-198

(61) Byford S et al British Journal of Psychiatry 2000; 176:537-543

(62) Byford S. et al Health Economics. 2007; 16(5):531-6.

(63) Flood C.et al British Medical Journal. 2006; 333(7571):729,

(64) Henderson C et al British Medical Journal 2004; 329, 136

(65) Dunn G et al Statistical Methods in Medical Research 2005; 14:369-395.

(66) Van Hout BA et al Health Economics 1994; 3 (5):309-319.

(67) Fenwick E et al Health Economics 2001; 10 (8):779-787.

(68) Davies S et al British Medical Journal 1996; 312: 533-537

(69) Morgan C et al British Journal of Psychiatry 2005; 186:281-289.

(70) Efron, B. et al Monographs on Statistics and Applied Probability No. 57 New York: Chapman and Hall/CRC 1994

# Appendix 1 – The CRIMSON Study Team

**Birmingham**

**Graham Thornicroft**

**CI**

Simone Farrelly

Scientific

Co-ordinator

RAs: Julia Cook & Catherine Finnecy

CSO: Juan Doblado Pavon

Admin: Lisa Moody

Max Birchwood

PI

**Manchester/Lancashire**

Max Marshall

PI Manchester

Richard Gater

PI Manchester

Waquas Waheed

PI Lancashire

**London**

Facilitator: Rangeni Zinyama

RAs: Adrine Woodham & Gillian Brown

CSO: Vicky Bell

Admin: Lisa Parkinson

Facilitator: Betina Reid

RAs: Samantha Treacy & Liz Doherty

CSO: Zoe

Admin: Joe Mirza

Facilitator: Melanie De Castro

**Health Economics/**

**Policy**

**Statistics**

**Reliability/**

**Fidelity**

**Focus Groups**

**Facilitators/ JCP**

**Sites**

**Focus Groups Service User & Trust**

**Data Manager**

Claire Henderson

Helen Lester

Diana Rose

Graham Dunn

Morven Leese

Sarah Byford

Martin Knapp

Kim Sutherby

Mauricio

Moreno

George Szmukler

PI

# Appendix 2 – Contact List

## Contact List for the CRIMSON Team

| **Name** | **Role** | **Email** |
| --- | --- | --- |
| Adele Fairbrother | PA for Max Marshall | adele.fairbrother@lancashirecare.nhs.uk |
| Adrine Woodham | RA Lancashire | adrine.woodham@lancashirecare.nhs.uk |
| Vicky Bell | CSO Manchester | Vicky.Bell@mhsc.nhs.uk |
| Barbara Barrett | Health Economist | Barbara.Barrett@iop.kcl.ac.uk |
| Betina McAlone | Facilitator Lancashire | Betina.Reid@lancashirecare.nhs.uk |
| Claire Henderson | Fidelity | Claire.henderson@iop.kcl.ac.uk |
| Diana Rose | Service User engagement, Focus Groups, | d.rose@iop.kcl.ac.uk |
| Samantha Treacy | RW London | Samantha.Treacy@kcl.ac.uk |
| George Szmukler | PI London | g.szmukler@iop.kcl.ac.uk |
| Gillian Brown | RA Lancashire | gillian.brown@lancashirecare.nhs.uk |
| Graham Dunn | Statistician | graham.dunn@manchester.ac.uk |
| June Flanagan | PA for Max Birchwood | June.Flanagan@bsmht.nhs.uk |
| Graham Thornicroft | Chief investigator | g.thornicroft@iop.kcl.ac.uk |
| Helen Lester | Focus Groups | helen.lester@manchester.ac.uk |
| Joe Mirza | Admin London | [Joe.mirza@iop.kcl.ac.uk](mailto:Joe.mirza@iop.kcl.ac.uk) |
| Kim Sutherby | Facilitator Trainer/supervisor | [kim.sutherby@slam.nhs.uk](mailto:kim.sutherby@slam.nhs.uk) |
| Lisa Parkinson | Admin Lancashire | [Lisa.Parkinson@lancashirecare.nhs.uk](mailto:Lisa.Parkinson@lancashirecare.nhs.uk) |
| Liz Doherty | RW London | Liz.Doherty@iop.kcl.ac.uk |
| Martin Knapp | Health Economist | sptemrk@iop.kcl.ac.uk |
| Mauricio Moreno | Data Manager | Mauricio.Moreno@iop.kcl.ac.uk |
| Max Birchwood | PI Birmingham | m.j.birchwood.20@bham.ac.uk |
| Max Marshall | PI Manchester/Lancashire | mmarshall@man.ac.uk |
| Melanie De Castro | Facilitator London | Melanie.DeCastro@iop.kcl.ac.uk |
| Morven Leese | Statistician | morven.leese@iop.kcl.ac.uk |
| Julia Cook | RA Birmingham |  |
| Rangeni Zinyama | Facilitator Birmingham | Rangeni.Zinyama@bsmhft.nhs.uk |
| Sarah Byford | Health Economist | s.byford@iop.kcl.ac.uk |
| Catherine Finnecy | RA Birmingham |  |
| Simone Farrelly | Scientific Co-ordinator | simone.farrelly@iop.kcl.ac.uk |
| Juan Doblado Pavon | CSO Birmingham | Juan.DobladoPavon@bsmhft.nhs.uk |
| Wendy Lamb | PA for Graham Dunn | [wendy.J.Lamb@manchester.ac.uk](mailto:wendy.J.Lamb@manchester.ac.uk) |
| Waquas Waheed | PI Manchester/Lancashire | waquas.waheed@lancashirecare.nhs.uk |

# Appendix 3 – Recruitment Plan

# Appendix 4 – Risks to blinding

Blindness to patient treatment allocation is an important methodological aim of this trial. The following procedures will be used to ensure that blindness is maintained throughout the trial.

**Randomisation**

1. It is the responsibility of the Research Workers to collect all baseline data from participants and electronic patient systems ***before*** alerting the Coordinator that the participant is ready to be randomised.
2. Research Workers will not be involved in the randomisation of subjects, other than alerting the Coordinator that a participant is ready to be randomised. After fully completing the baseline assessment interview, Research Workers will inform Simone Farrelly via the dedicated form in the data entry database that the participant is ready to be randomised. SF will then access the online randomisation service hosted by the IOP CTU and enter in the participants details. The Research Workers will receive an email alerting them to the fact that their participant has been randomised. At this point, the Research Worker will then enter that they have received confirmation of randomisation in the data entry database. The baseline data for this individual will then be blocked.
3. After the randomisation confirmation has been received by the Research Worker and they have entered this into the data entry database, Research Workers should not look at the participants clinical notes again.
4. The JCP facilitator at each site will receive an email informing them that a participant has been randomised and to which intervention arm. It is the responsibility of the facilitator at each site to ensure that the Research Workers do not see this correspondence.
5. The facilitator at each site will then inform both the control and intervention groups of the allocation. It is the responsibility of the facilitator to ensure that the Research Workers do not see this correspondence.
6. When discussing the allocation with the participant, the facilitator will give the participant the SF’s or local Trial administrator’s number in case he or she wishes to discuss the allocation further at a later date.
7. Facilitator to file randomisation notification emails and other identified information regarding intervention arm, in locked filing cabinet separate to Research Worker files

**Intervention**

Facilitators and local site lead PI have a responsibility to ensure that Research Workers do not discover which participants receive the intervention by:

1. Ensuring that Research Workers are using separate offices, phone numbers, file storage to the facilitators.
2. Ensuring that team research meetings do not involve discussion of the participants, even in casual conversation. Team meetings may be conducted with a portion that is not attended by the research workers in which any specific participants can be discussed.
3. Facilitators should remind the participant at the JCP and content checking meetings, and at any subsequent contact with the participant that the Research Worker should not know the group allocation. Family members and clinical staff should also be reminded that blindness must be maintained.
4. If possible, Research Workers should inform the facilitators of their upcoming assessments (e.g. in meeting minutes or passing the appointment information to the administrator) so that facilitators can avoid being in the same location at the same time for the JCP meetings and content checks.
5. Where possible and safe, Research Workers should conduct assessments at participants homes or places other than the CMHT building.

**Follow-up assessments**

1. Research Workers also have the responsibility to remind participants/family members and clinical team that blindness must be maintained – this is particularly important at the follow-up assessment. To this end,
   1. Standard letters will be sent to participants (from the contacts database) during the follow-up period with a newsletter, Christmas cards, alerting them to the fact that their baseline assessment is due, confirming their appointment details etc which state, that the Research Worker cannot know if the participant developed a JCP or not.
   2. When phoning and at the assessment, the Research Worker should restate that they cannot know if the participant developed a JCP or not.
2. Site leads are responsible for ensuring that data that needs to be collected from electronic systems/case notes (admissions and contact data) shall be requested in electronic form from centralised IT systems. RW should not access electronic systems at the follow-up assessment as the JCP will be attached and their may be a flag/alert regarding the presence of a JCP.
3. Where feasible, the administrator’s number should be given out to participants instead of the Research Workers or facilitator’s individual numbers. The administrator can then direct messages and this will help to avoid situations where a participant mistakenly calls the Research Worker looking for the facilitator.

**In case of blind breaches:**

1. SF and the site lead should be notified via email
2. SF will complete a form to record the breaching
3. A different Research Worker at the site will take over and complete the assessments Where this is not possible, the unblinded Research Worker should co-rate with SF.

**Success of blinding**

At follow-up, each Research Worker will be asked to guess which intervention arm a participant is in.

# Appendix 5 – Analysis Plan.

**Analysis Plan Draft 2**

**8/26/2008**

Individual-level single-blind RCT of Joint Crisis Plans (JCPs) compared with a ‘treatment as usual’ control condition for people with a history of relapsing psychotic illness in Birmingham, London and Manchester. There will be one follow up point at 18 months after the completion of the JCP.

Allocation will be by randomised permuted blocks of randomly varying block size (sizes 2 and 4), with equal allocation to the two arms, stratified by centre. The Mental Health & Neuroscience Clinical Trials Unit (MH&N CTU) will perform the allocations.

**The total to be recruited is 540, or 180 per site (which includes 48 who also have qualitative assessments after the 18 month follow-up).**

**Primary Outcome**

Proportion of service users admitted or otherwise subsequently detained under an order of the Mental Health Act during the follow-up period.

Sample Size

**The primary outcome is reduction in the proportion of service users admitted or detained under a Mental Health Act section at least once, obtained from records. In the pilot trial based in London 26% were compulsorily admitted over 15 months, equivalent to 30% over 18 months. Routine data for inner city wards in Birmingham and Manchester show a very similar proportion, on average. Assuming that a clinically important reduction would be to at least halve the proportion, i.e. a reduction in absolute terms by 15% to 15%, 90% power using a double-sided test with alpha=0.05 would require 174 in each arm.**

**For the ethnic subgroup analysis where the baseline compulsory admission rate is likely to be higher, an achieved subsample of 91 per arm would give 80% power to detect a difference from 40% to 20% Given the percentages of service users likely to be black at each site (from recent actual MHA use data), 90 are likely to be found with a sample of 270 per arm, and the minimum achieved would be about 80 with a slight reduction in power.**

**Loss to follow-up is likely to be about 15% for the interview data so this sample size (270) would reduce to an effective 229 per arm, which would allow standardised effect sizes of 0.3 for the secondary outcomes to be detectable with 90% power. The total to be recruited would therefore be 540, or 180 per centre.**

**Measures**

B=Baseline, FU Follow-up at 18 months post baseline

**Colleagues with as special interest in the outcome scales are noted below in case of queries regarding scoring or interpretation. This is a preliminary suggestion and subject to change – please contact SF or ML if you prefer not to be the ‘expert’ for the particular scale or would rather take on another (or no) scale.** **We need someone to take an interest in the Engagement and Acceptance scale**

| **Primary/secondary outcomes** | **Standardised measure** | **Source** | **Time** |
| --- | --- | --- | --- |
| MHA use (primary) | Routine hospital datasets | Admin. Records + Patient Information Records using Admissions Proforma* | B FU |
| Informal admissions | As above | As above | B FU |
| Recovery Style  (MB) | Recovery Style Questionnaire | Self-report** | B FU |
| Perceived coercion  (GS) | Treatment Experience Survey (Community) | Self-report (based on MacArthur AES) ** | B FU |
| Engagement with care  (?) | Engagement and Acceptance Scale | Staff-rated | B FU |
| Therapeutic relationship  (JS) | Working Alliance Inventory | Self-report**  and Staff-rated | B FU |

*To be collected for all patients including dropouts (refusals or uncontactable) unless patient specifically requests otherwise.

** Interviewer helps in cases of literacy problems only

| **Other outcomes*** | **Standardised measure** | **Source** | **Time** |
| --- | --- | --- | --- |
| Functioning  (SF) | Global Assessment of Function (GAF) | Rater | B FU |
|  |  |  |  |
| Involuntary transport to hospital  under the MHA | Admissions Proforma | Case notes | B FU |
| Police involvement in admission | Admissions Proforma | Case notes | B FU |
| Incidents of violence and sef-harm  (MB?) | COMMAND questionnaire | Interview | B FU |
| Criminal justice system involvement  (SB/BB) | Crimson AD-SUS | Interview | B FU |

Is GAF an outcome as such or is it a predictor?

| **Socio-demographic data*** | **Standardised measure** | **Source** | **Time** |
| --- | --- | --- | --- |
| OPCRIT diagnosis  (MHRN CSO at each centre) | OPCRIT | Case notes | Eligibility (prebaseline) |
| Centre |  |  | B |
| Gender | Crimson Sociodemographics | Interview | B |
| Ethnic group (W, BA, BC, other) | Crimson Sociodemographics | Interview | B |
| Age | Crimson Sociodemographics | Interview | B |
| Clinical diagnosis  (SF) | Crimson Sociodemographics | Case notes (text -codes to be decided) | B |
| Educational level | Crimson Sociodemographics | Categories to be combined | B FU |
| Marital Status | Crimson Sociodemographics | Categories to be combined | B FU |
| Accommodation (lives with, type) | Crimson AD-SUS | Categories to be combined | B FU |
| Occupational classification (ONS) | Crimson AD-SUS | Categories to be combined | B FU |
| Employment | Crimson AD-SUS | Categories to be combined | B FU |

* Key variables only included here

**Further data**

- Fidelity and Quality Rating of Plan Scores

Fidelity score A (preparatory meeting) and B (joint crisis planning meeting), rated from audio recordings by KS and CH.

Currently 10 per centre. But if scores fro clinical and non clinical facilitators to be compared then much larger sample required (suggest all) and interrater reliability needs to be considered.

Quality Rating of Plan – to be finalised by ?

- Activation of Plan

Whether or not plan actually activated and/or paid attention to? How to collect without affecting intervention or blinding, resource issues, type of data that is available, consistency across centres.

- Care Coordinators

Data to be collected on care coordinators will be gender, ethnicity, age, professional qualification (OT, CPN, psychologist, other), length of relationship with participant, length of practice, first language (NB patient’s first language not collected – collect at followup?).

**Data Management**

The package MACRO (via the IOP CTU) will be used for data input. Interim reports on numbers recruited (including ethnic breakdown by arm) and random data quality checks will be made (all primary outcome and 10% of others to be checked by SF) every quarter.

**Type of analyses**

An intention-to-treat analysis will be applied in the first instance (i.e. analysing all available data from service users in their groups as randomised), using double-sided critical values at 0.05.

No adjustment for multiple testing but secondary analyses will be treated as exploratory.

There will be no interim analyses. All hypotheses-related analyses will be conducted only at the end of the follow-up period.

**Statistical Methods**

The principal analysis will compare the primary and secondary outcome measures at 18 months, combined over centres. The proportions admitted to hospital under a section at follow up will be compared between randomisation groups using logistic regression controlling for centre and ethnic group.

Other (continuous) outcomes such as therapeutic alliance and engagement with mental health services will be analysed using analysis of covariance controlling for baseline (pre-intervention) measures, ethnic group and centre. Short scales will be analysed using ordered logistic regression, otherwise linear regression. Bed-days and other very skewed data will be analysed using bootstrapping to obtain confidence intervals and p-values.

Number of admissions (informal and under MHA) will be analysed using Poisson regression, and time to first admission using survival analysis

SPSS version 12 and Stata version 10 will be used for the analyses.

**Missing Data**

Some outcome data (in particular the primary outcome) will be obtained from staff, case notes, and administrative sources, and even for service users lost to follow-up.

Sensitivity analyses will be performed to assess the influence of loss to follow-up and refusals, including imputation of missing baseline values from the within-centre means; multiple imputation of follow up values (where feasible from other variables) and controlling for any baseline variables that are related to missing outcomes in the analysis.

- The content of the facilitators’ meetings will be assessed using the specially designed fidelity scale.
- The content of the Care Programme Approach of randomly selected cases in both arms will be tested at 9 months.

Number to be decided – probably on the basis of an equivalence test on some total score depending on how this is measured (scale to be developed).

- Research raters’ ‘best guesses’ of trial arm will be assessed to test whether their blindness is maintained*.*

**Planned Subgroup Analyses**

- Ethnic group (Whites, the combined Black subgroup, which consists of black Caribbeans and black Africans, and others).

The study is not specifically powered to detect interactions between ethnic group and treatment arm. However the black group is considered to be of interest in its own right and therefore a parallel analysis will be applied to this subgroup. The purpose would not be to identify differences compared to the white group but to separately establish the effectiveness of the intervention for black clients.

- Facilitators: the scope for analysing their individual effect will be limited (although see above under fidelity: the fidelity score for particular facilitator/user meetings will be included in further exploratory analyses of intervention effects and also to compare clinical facilitators with their nonclinical backups.
- Care coordinators: variation contributed by care coordinators will be taken account of by including them as random effects in regression models, in which the effect of their characteristics will also be assessed.

Appendix 6 - Publication Protocol

**Common data set**

1. All data from the study, including that held on a local database at any site, is part of a common data set unless agreed otherwise at a steering group meeting. Participants in the project include the steering group, anyone employed to take part in the project, and anyone who is given access to the data set by the steering group for the purpose of preparing results for publication.

2. Data management will be co-ordinated from the London site, where a final data set will be assembled.

3. Data entry and cleaning will be the responsibility of each centre.

4. We shall agree variable names and definitions prior to data entry and not modifying these without agreement.

**Criteria for Authorship**

5. The names in the authorship and their sequence should reflect relative contribution to the writing of the paper as well as the analysis, design and conduct of the study. It may be appropriate for some contributions to be recognised by an acknowledgement.

6. The research group will take into consideration the authorship section of the Uniform Requirements for Manuscripts Submitted to Biomedical Journals^1^. The requirements state:

*‘All persons designated as authors should qualify for authorship. Each author should have participated sufficiently in the work to take public responsibility for the content.*

*Authorship credit should be based only on substantial contributions to 1) conception and design, or analysis and interpretation of data; and to 2) drafting the article or revising it critically for important intellectual content; and on 3) final approval of the version to be published. Conditions 1,2, and 3 must all be met. Participation solely in the acquisition of funding or the collection of data does not justify authorship. General supervision of the research group is not sufficient for authorship. Any part of an article critical to its main conclusions must be the responsibility of at least one author.*

*Editors may ask authors to describe what each contributed; this information may be published.*

*Increasingly, multi-center trials are attributed to a corporate author. All members of the group who are named as authors, either in the authorship position below or in a footnote, should fully meet the above criteria for authorship. Group members who do not meet these criteria should be listed, with their permission, in the Acknowledgements or in an appendix.*

*The order of authorship should be a joint decision of the co-authors. Because the order is assigned in different ways, its meaning cannot be inferred accurately unless it is stated by the authors. Authors may wish to explain the order of authorship in a footnote. In deciding on the order, authors should be aware that many journals limit the number of authors listed in the table of contents and that the U.S. National Library of Medicine (NLM) lists in MEDLINE only the first 24 plus the last author when there are more than 25 authors.’*

**Acknowledgements**

7. Publications shall mention ‘MRC-funded’ study.

8. An acknowledgement paragraph shall be included (to be completed) as follows:

9. Authors will need to assess whether additional acknowledgements are appropriate for individuals who have been of particular help in individual papers.

**Procedures of writing and submitting a publication**

10. All members must adhere to the publication plan, regarding authorship, content and target journal.

11. The first author will take the responsibility of writing the first draft of the paper and will be responsible for circulating the paper to all other authors. The first author will collect comments from co-authors and make appropriate amendments. The final draft of any paper must be circulated to all authors, who should be in agreement with its content before it is submitted for publication. Consent should also be sought from those whose contribution has been acknowledged. Written agreement regarding content and target journal must be obtained before the first submission for publication.

12. All papers must state the affiliation to the wider research collaboration. All submitted papers are to be sent to the co-ordinating centre which will make them available on request to other sites.

**Presentations**

13. In view of the risk of prior publication, there will be no presentations based on the common data set prior to publication of the key papers*.* In addition, numerical data of other kinds such as recruitment rate or details of CONSORT diagrams should not be published in advance of the key papers.

14. While conference presentations in advance of publication of the key papers may be of value in publicising the study, numerical data of any kind such as recruitment rate or details of CONSORT diagrams should be generalised and approximated to minimise the risk of prior publication and should not be permitted to be published in any form of publication including conference abstracts. Planning of major conference presentations will be included in the agenda of the steering group meetings.

15. A standard slide with acknowledgements will be prepared.

16. When a public presentation of data is to be made and data is taken from a paper already accepted for publication, collaborating authors must be acknowledged. Where the data for presentation is taken from a paper still in preparation, permission must be obtained from the relevant authors for their findings to be presented and the authors likewise acknowledged.

**Publication plan and possible authors**

17. A list of agreed publications, the publication plan, will be developed by the completion of recruitment to the study and will be tabled and agreed at each subsequent meeting. This will identify the first author, the subject of the paper, and the data on which it is to be based. Papers should be planned as early as possible to avoid content overlap and ensure fair allocation of authorship.

18. Individuals who wish to propose a paper based on shared data should circulate proposals to all participants, giving a specified time period in which to respond to the proposal. If no objections are received the paper will be added to the publication plan to be ratified at the next steering group meeting.

19. Co-authors may also be agreed at a steering group meeting, or the meeting may agree that the invitation of co-authors should be left to the first author or to site leads (subject to the criteria for authorship given above).

20. Amendments to the publication plan will, as a rule, be agreed at steering group meetings. Under exceptional circumstances, new papers can be agreed between meetings. Such suggestions will be circulated to all members of the steering group, who can raise objections within a month.

21. The first author of any paper which is included in the list of deliverables for a particular site, or any other paper resulting from work which is entirely within the workpackage of a particular site will be from that site unless agreed otherwise by the lead for that site. The process of selecting co-authors will then be a matter for individual sites, subject to the statement on authorship criteria above.
